# *In Situ* Representations and Access Consciousness in Neural Blackboard or Workspace Architectures

**DOI:** 10.3389/frobt.2018.00032

**Published:** 2018-04-03

**Authors:** Frank van der Velde

**Affiliations:** Cognitive Psychology and Ergonomics, Institute for Digital Society, University of Twente, Enschede, Netherlands

**Keywords:** access consciousness, connection paths, global workspace, *in situ* representations, neural blackboard architectures, robots

## Abstract

Phenomenal theories of consciousness assert that consciousness is based on specific neural correlates in the brain, which can be separated from all cognitive functions we can perform. If so, the search for robot consciousness seems to be doomed. By contrast, theories of functional or access consciousness assert that consciousness can be studied only with forms of cognitive access, given by cognitive processes. Consequently, consciousness and cognitive access cannot be fully dissociated. Here, the global features of cognitive access of consciousness are discussed based on neural blackboard or (global) workspace architectures, combined with content addressable or “in situ” representations as found in the brain. These representations allow continuous cognitive access in the form of a process of covert or overt queries and answers that could underlie forms of access consciousness. A crucial aspect of this process is that it is controlled by the activity of the *in situ* representations themselves and the relations they can initiate, not by an external controller like a CPU that runs a particular program. Although the resulting process of access consciousness is indeed based on specific features of the brain, there are no principled reasons to assume that this process cannot be achieved in robots either.

## 1 Introduction

In one sense, discussing consciousness in a (humanoid) robot is easier than discussing human consciousness. In the latter case, we are hampered by our own “first-person” perspective. We “know” what it means to be conscious because we experience it ourselves. However, this first-person perspective is a form of introspection, which is out of reach for scientific observation and discussion.

The influence of the first-person perspective is clear in the distinction between two different views on the nature of consciousness, known as phenomenal consciousness and functional (or access) consciousness (Block, 1995; Cohen and Dennett, 2011; Taylor, 2012). Phenomenal consciousness asserts that conscious experiences result from specific neural correlates in the brain. Examples of these, depending on the theory at hand, are recurrent connections in the brain (e.g., Block, 2007), specific “microactivations” distributed over the brain (Zeki, 2003), or “winning” coalitions of neurons that result in a conscious experience of the representation they instantiate (Crick and Koch, 2003). The key notion of phenomenal consciousness is that the neural correlates responsible for consciousness can be separated (dissociated) from all the cognitive functions we can perform, such as attention, language, and the like. That is, consciousness “overflows access” (Cohen and Dennett, 2011).

For robotics, this would mean that the search for robot consciousness is doomed. Unless we endow robots with the required neural correlates (as in hybrid forms of neuro-robots), robots cannot possess forms of consciousness.

However, as convincingly argued by Cohen and Dennett (2011), phenomenal consciousness is a view on consciousness that is outside the reach of science precisely because it assumes neural correlates of consciousness separate from neural correlates of cognitive functions. A consequence of this view is that theories of consciousness cannot be empirically verified or falsified (which would always depend on some form of behavior produced by some kind of cognitive process).

By contrast, theories of functional or access consciousness assert that consciousness can be studied only with forms of cognitive access, given by cognitive processes. Consequently, consciousness and cognitive access cannot be fully dissociated. Instead, any form of consciousness would require a cognitive architecture that would allow forms of functional access.

An influential proposal for such an architecture is the Global Workspace theory, which asserts that consciousness arises when representations enter the Global Workspace of the brain and (temporarily) one of them dominates its activation (e.g., Baars, 2002; Baars and Franklin, 2003; Wiggins, 2012). The perspective I address here is that this theory could indeed provide the basis for a cognitive and computational architecture for functional consciousness, provided it is combined with a key observation on the nature of representation in the brain. This observation concerns the notion that representations in the brain are “*in situ*,” which entails that they operate (at least in part) always as the same representation in each instantiation of the cognitive processes in which they participate.

In this way, *in situ* representations differ fundamentally from representations as used in von Neumann architectures, in which representations are inert, stored in arbitrary locations under the control of a CPU. By contrast, cognitive processes based on *in situ* representations are controlled by these representations, and not by an outside controller like a CPU that runs a particular program. This could result in a continuous process of “queries and answers” (van der Velde, 2013), which could form the basis for forms of access consciousness.

In the following sections, I will describe the notions of *in situ* representations, functional consciousness, and their relation in more detail.

## 2 *In Situ* Representations

A striking feature of representations in human cognition, as argued here, is their content-addressable nature. In this way, a representation can be (re)activated by directly activating it or a part of it. This is different from a representation in computers, which is accessed by means of its address label (which is also true for files in Github, where address labels are derived from the content of the file). In this case, a list of address labels needs to be run through first to find the label.

The notion of content-addressable representation is at the basis of many theories of human semantic representation (but see below for a counter example) and was one of the main motivations for the rise of connectionism in the 1980s (Bechtel and Abrahamsen, 1991). For example, Hebb (1949) used content addressability as the basis for his notion of the “cell assembly” hypothesis of (concept) representations in the brain. According to this hypothesis, a cell (or neural) assembly of a concept develops over time by interconnecting those neurons in the brain that are involved in processing information and generating actions related to that concept. These assemblies could be distributed over (very) different parts of the cortex (and other brain structures), depending on their nature.

A more recent version of a similar model of content-addressable representation in the brain is the “hub and spoke” theory of semantic representation in the brain (Lambon Ralph et al., 2017). In this theory, based on behavioral and imaging studies, modality-specific semantic information is represented in brain areas that process that kind of information (e.g., visual information in the visual cortex and auditory information in the auditory cortex). These kinds of representations are the “spokes” of semantic representations in the theory. However, the spokes are interconnected in (bi-lateral) hubs located in the anterior temporal lobes. Hub representations are transmodal, in that they respond to and correspond with cross-modal interactions of modality-specific information. Examples of transmodal representations in the temporal cortex were also observed in single-cell studies with human subjects. For example, neurons were found that responded to (the identity of) a person, regardless of whether the face of the person (visual information) or name (visual or auditory information) was presented (Quian Quiroga, 2012).

A crucial point here is that transmodal hub representations interconnect the modal spoke representations. But they do not replace or stand in for them. That is, their content is determined by the spoke representations they are connected to, and that content is reactivated when the hub representation is activated. This is what the cell assembly idea of Hebb is about. It is also in agreement with the imaging (fMRI) observations of Huth et al. (2016), who, in an extensive study, measured brain activity related to words when people were listening to stories. So, auditory language information was presented, but it activated a large set of cortical areas that responded to (also modal) semantic information, both in the left hemisphere (63 semantically selective areas) and the right hemisphere (77 semantically selective areas) of the cortex.

Hence, even though representations can have parts in transmodal hubs, they consist of a (potentially) large set of neurons (an assembly) distributed over widely different areas in the cortex, depending on their content. This shows why they are content addressable. By activating, say, the hub part of a representation, its spokes will be activated as well (as in the Huth et al. (2016)), revealing the content of the representation. But when (a part of) the spokes are activated by, for example, perceptual information, the hub part and consequently the other spokes can be activated as well. So, each activation of a representation potentially entails the activation of the entire hub and spokes. Crucially, this will be the same hub and set of spokes for each new activation of the representation (which, or course, can develop and change over time).

This is why these representations can be referred to as “in situ” (van der Velde, 2016). They do not consist of some (neural) code that can be copied and transported elsewhere, but of the entire web-like hub and spoke structure (which would be impossible to copy and transfer to somewhere else in the brain, e.g., given its distributed nature).

## 3 Computational Architectures Based on *In Situ* Representations

The nature of *in situ* representations in the brain raises the question of how they function in cognitive processes. In particular, in productive forms of cognitive processing, because these would seem to be the kind of cognitive processes that are needed to test forms of functional consciousness (Cohen and Dennett, 2011).

Productive processing entails that information is processed or produced in a combinatorial manner, based on (more elementary) constituent representations (concepts) and their relations. Productive processing is of key importance for human cognition, as found in language, reasoning, and visual perception. Consequently, they can be expected to play a key role in consciousness as well, as in relating conscious experiences to each other (e.g., *the apple is red* versus *the apple is green*).

Combinatorial processing with representations that are not copied but remain *in situ* can be achieved in architectures that provide (temporal) connection paths between the constituent representations, in line with their relations. For example, consider the combination *red apple*, with *in situ* representations for *red* and *apple*. Each one consists of an assembly structure with spokes in parts of the cortex related to perception or actions, such as seeing or eating an apple, and links to the transmodal hub in the anterior temporal cortex.

**Figure 1:**
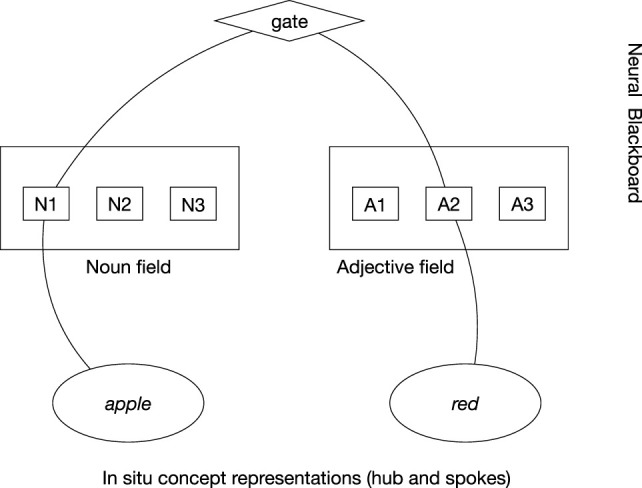
*In situ* representation of *red apple* by a connection path between *in situ* concept representations in a neural blackboard architecture. The noun *apple* first binds to a Noun assembly (here, N1) in the Noun field of the neural blackboard and *red* binds to an Adjective assembly (here, A2) in the Adjective field. The connection path passes through gates, which provides control to represent relations. Here, activation of “gate” gives adjectives bound to nouns, here *red* to *apple*.

In the neural blackboard architecture of van der Velde and de Kamps (2006), the relation *red apple* is produced by establishing a (temporal) connection path between the *in situ* concept representations of *red* and *apple*, as illustrated in Figure 1. The path is achieved in a “neural blackboard,” which could be connected in particular to the hub part of the *in situ* representations.

In this neural blackboard, the concepts are temporarily bound to “structure assemblies” in line with their word type. So, *apple* is bound to a “Noun assembly” in a “Noun field” and *red* is bound to an “Adjective assembly” in an “Adjective field.” Such word type fields are in line with the existence of (agent and object) areas in the (temporal) cortex that are selectively activated when nouns function as agents (subjects) or objects of verbs (Frankland and Greene, 2015). In turn, the Noun assembly bound to *apple* and the Adjective assembly bound to *red* can be temporarily bound to each other, representing the relation *red apple*. The structure of the neural blackboard is such that it allows the combination of arbitrary words in a familiar language (van der Velde and de Kamps, 2015).

Neural blackboards would not only exist to process or produce conceptual structures (e.g., relations between words in a sentence) but also, for example, to process relations between visual features, as found in the structure of the visual cortex. Here, I am not discussing the specific way in which conceptual or visual relations can be processed in terms of *in situ* representations (see van der Velde and de Kamps (2006) for detailed descriptions), but the consequence of this form of representation for cognition, and potentially for functional consciousness, as outlined in the next section.

## 4 Functional Consciousness

The relation between *in situ* representations and functional consciousness, and they way they differ from phenomenal consciousness, can be illustrated with a “perfect” experiment described by Cohen and Dennett (2011). Assume we have a subject in which the area in the brain responsible for color consciousness is isolated from other brain areas higher up in the activation stream. So, this area (say V4 or inferotemporal cortex) would receive feedforward input from lower areas in the visual cortex, as in the normal situation, but cannot generate output to other areas. When a colored object is presented, say a red apple, the color area would be activated by and in correspondence with the color of the apple, as in the normal case. But activation of the color area itself is isolated from the rest of the brain.

Because of its isolation, the color area would not, for example, activate brain areas underlying language anymore. So, when presented with a red apple, our subject would not be able to say that the color is red. Indeed, she could not indicate by any form of action what the color of the apple is. Also, she would not become emotionally affected by the color (if that was the case before the isolation) because these areas are not activated by the color area anymore either. In fact, she would indicate that the apple is colorless. Yet, according to phenomenal consciousness theories, our subject would still be conscious of the color red, as its neural correlate is active. This activity could also be measured by brain imaging, supporting the notion that our subject is in fact conscious of red, even though she indicates by any form of action or emotion that she is not.

Thus, although our subject would not (and could not) indicate that she has a first-person experience of red, theories of phenomenal consciousness would still assert she has. But this assertion is untestable, because no action of our subject can indicate that she is conscious of it. Consequently, the theory that supports such a form of consciousness is unverifiable. To make a theory of consciousness verifiable, some form of action is needed to identify an experience as conscious. Hence, consciousness and cognitive functions are not fully dissociated. This is the notion underlying functional (or access) consciousness (Cohen and Dennett, 2011).

The “perfect” experiment of Cohen and Dennett (2011) relates directly to *in situ* representations because it entails that the *in situ* representation of, say, the concept *red* is broken. The *in situ* representation of this concept not just consists of the connections that activate it but also of the connections that activate related concepts and circuits that produce behavior related to the concept (as saying the word red or pointing to a red object in a display).

So, the integrity of an *in situ* representation, in particular its ability to produce behavior, is crucial for its role in functional consciousness. This raises a reverse question. Suppose a subject would produce behavior like saying that she is conscious of the color red. Is that sufficient to conclude she is? At face value, this would be the result of a theory of access consciousness, because the cognitive function of identifying the color would entail the conscious experience of it. In that case, all that would be required for robots to be conscious, say of colors, is their ability to indicate the color of an object, by speech or another cognitive function.

However, just saying “red” does not indicate what a subject is (fully) conscious of. Words are labels to indicate an experience or concept but often do not cover their entire content. This observation does not entail a form of phenomenal consciousness. It just indicates that more elaborate forms of access (other words, or other forms of action) are needed to unravel the content of a conscious experience.

To see how this could proceed, we need to look at the way in which *in situ* representations function in a Global Workspace architecture.

## 5 Consciousness Based on Queries and Answers with *In Situ* Representations

In the Global Workspace theory of consciousness, representations compete to get access to the workspace and to (temporarily) dominate it (e.g., Baars, 2002; Baars and Franklin, 2003; Wiggins, 2012). This raises the question of how representations can enter the workspace and how the domination of the workspace is related to consciousness. The notion that representations in the brain are *in situ* could provide the beginning of an answer to these questions. If so, the underlying architecture could also form a basis for robot consciousness.

An *in situ* representation would not “enter” the global workspace but instead would be connected to it, with a connection path as illustrated in Figure 1. If the workspace would have the structure of a neural blackboard as illustrated in this figure (or this neural blackboard would be a part of it), the “entrance” of a representation in the workspace would consist of a temporal activation of this connection path to and in the workspace. Several *in situ* representations could then compete, resulting in one representation (and its connection path) temporarily dominating the workspace.

The dominating *in situ* representation selected in the workspace could then form the basis for a functional form of consciousness by a (continuous) “process of explicit or implicit queries and answers” (van der Velde, 2013).

As an illustration, consider the entire representation of *red apple* in the neural blackboard architecture outlined in the previous section. Again, assume that a similar connection path would exist in the global workspace (or, alternatively, that the neural blackboard is a part of the workspace). Because of its *in situ* nature, the neural representation of the concept *red* would be connected to the visual areas in the brain that process and represent color, but also to the neural word representation *red* in language areas. The *in situ* representation of *apple* would be connected to the visual areas responsive to shape, and the word representation *apple* in the language areas.

The connection path between them in the neural blackboard (or global workspace) forms the basis for functional access and behavior, in which the relation between the *in situ* representations can be expressed in an action. So, for example, the (explicit or implicit) query “What is the color of the apple?” would be answered by activating the *in situ* representation of *apple* (e.g., by seeing it or hearing the word *apple* in an actual question) and the condition that allows the activation of Adjective assemblies bound to Noun assemblies (e.g., of *apple*) in the neural blackboard. In turn, this results in the activation of the *in situ* representation of *red* through the connection path that interconnects *apple* and *red* in the neural blackboard (or global workspace). This would form the basis for generating a response (reflecting functional access) such as pointing to the red object or reporting the word *red*.

The key notion of this process is that it is initiated and controlled by the *in situ* representations, and not by an outside controller like a CPU that runs a particular program. Hence, it will be a continuous process, in which activated *in situ* representations initiate queries to “ask” for other semantic information related to them (also represented by *in situ* representations). This continuous activation process underlies a continuous form of functional access, which in turn could be the basis for a process (stream) of access consciousness. More specific examples of this process and its relation to consciousness are presented in van der Velde (2013).

**Figure 2:**
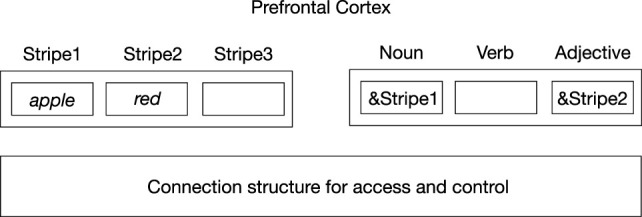
Indirection representation of *red apple* based on Kriete et al. (2013). Neural codes for concepts (e.g., *apple, red*) are stored in memory locations (“stripes”) in the prefrontal cortex. The addresses of these stripes (as given by the address operator &) are then stored in “role stripes,” needed to establish the relation between the concepts. An underlying connection structure provides and controls access to stripes.

The importance of the fact that this process is controlled by *in situ* representations is further illustrated in Figure 2. This figure illustrates an indirect way of representing content information in the brain, as given by the indirection model of Kriete et al. (2013). Here, neural codes of *red* and *apple* are (temporarily) stored in “stripes” located in the prefrontal cortex (PFC). The stripes operate as registers in a computer memory. In turn, their address can be stored in other PFC “role stripes” (here for noun and adjective), which represents the relation *red apple*. So, the query *apple color?* can be answered by first retrieving the role stripe (here, noun-stripe) that stores the stripe address of *apple* (&Stripe1) and then going to the adjective stripe related to that noun-stripe. Then, the address code of the adjective (&Stripe2) can be retrieved, which will result in finding the location (stripe) where the neural code for the color (*red*) is stored.

In this process, the representations themselves are inactive and not content addressable. They can be retrieved only by finding the addresses of the locations where they are stored. These addresses can be different on different occasions, depending on the preceding representations stored in the process. As a result, the content of a given address (stripe) can vary from occasion to occasion. Hence, content and address are dissociated. So, activation of an address itself gives no information about its content and therefore cannot play a direct role in access consciousness.

Furthermore, a content code (e.g., of *red*) is generally not accessible when it is stored in a given stripe. For example, access to that given stripe needs to be blocked when other content is to be stored in other stripes. Otherwise, the content of the given stripe could inadvertently be deleted (overwritten) in the process of storing other representations in other stripes. Hence, the representation of *red* in Figure 2 resembles the isolated color representation in the perfect experiment of Cohen and Dennett (2011) discussed earlier. It may be active within the stripe, but its access to processes outside the stripe, and hence its active involvement in these processes, is generally blocked. In other words, the content representations are generally inactive because the stripes in which they are stored are generally “closed.”

The indirection model of Kriete et al. (2013) is a model for productive computing in the brain, closely resembling productive computing in a Von Neumann architecture. So the in-activeness (and in-accessibility) of representations and their (negative) consequences for functional or access consciousness as discussed earlier would also hold for the Von Neumann architecture, which underlies digital computing. In turn, digital computing still forms the basis of many robot systems, such as the iCub robot (Natale et al., 2016).

So, the analysis of access consciousness as given here would have consequences for robot consciousness as well. In particular, it would seem that forms of robot consciousness would require a computing architecture based on *in situ* computing as illustrated above, instead of the Von Neumann kind of architectures still used to date. If correct, robot consciousness would indeed be based on specific features of the brain. But, in contrast to the assertion of phenomenal consciousness, it would not be based on specific physiological features of the brain, most likely unobtainable for robots, but on its specific computing and cognitive architecture.

## 6 Conclusion about Robot Consciousness

The analysis presented here provides a few suggestions about the possibility and requirements of robot consciousness. First, consciousness seems to be related to *in situ* representations that underlie the possibility of cognitive access. Second, consciousness is more a process than a set of isolated conscious states. This, in combination with the requirement of access, suggests that consciousness is related to a continuous process of cognitive access. Third, this continuous process does not take the form of isolated instances of indirect activation of representations under the control of an external controller. Instead, the perspective is offered here that a continuous process of access can be achieved only when the process is directly controlled by (the activity of) *in situ* representations themselves, as in a continuous (covert or overt) process of queries and answers. Such a process seems to be in accordance with cognitive processing and access consciousness as found in the human brain. There are no principled reasons to assume that this process cannot be achieved in robots either.

## Author Contributions

FV conceived and wrote the article.

## Conflict of Interest Statement

The author declares that the research was conducted in the absence of any commercial or financial relationships that could be construed as a potential conflict of interest.
